# NuViva: Development of postpartum nutrition and exercise program implementing the FIGO pregnancy passport to reduce cardiometabolic risk

**DOI:** 10.1002/ijgo.70877

**Published:** 2026-02-20

**Authors:** Nikki M. W. Lee, So‐Ling C. Lau, Panda Hoi Yin Li, Kit Ying Tsoi, Liona C. Poon

**Affiliations:** ^1^ Department of Obstetrics and Gynecology, Prince of Wales Hospital The Chinese University of Hong Kong Hong Kong SAR China; ^2^ PhysioMotion Limited Hong Kong SAR China; ^3^ Danone Nutricia Early Life Nutrition Hong Kong SAR China

**Keywords:** adverse pregnancy outcomes, cardiometabolic risk, FIGO Pregnancy Passport, gestational diabetes mellitus, hypertensive disorders of pregnancy, lifestyle intervention, long‐term health, postpartum care

## Abstract

Pregnancy provides a unique opportunity to identify women at increased risk of future chronic disease, as adverse pregnancy outcomes (APOs) such as hypertensive disorders of pregnancy, gestational diabetes mellitus, preterm birth, placental complications, and fetal growth restriction are associated with later cardiometabolic morbidity. However, postpartum care remains fragmented and limited, representing a missed opportunity for preventive intervention. Currently in pilot phase, NuViva is a novel, multidisciplinary, community‐based postpartum program, providing nutrition, physical exercise, and pelvic floor assessment education at 4 time points over a 12‐month period post delivery. A smartphone application, based on recommendations of the FIGO pregnancy passport, supports biometric record keeping, self‐monitoring, education, and adherence. Assessments include blood pressure and biometrics; laboratory investigations where indicated; pelvic floor assessment; functional fitness tests; and validated psychosocial, lifestyle, and nutrition questionnaires. The primary outcome is cardiovascular health score at 1 year postpartum. Secondary outcomes include anthropometric measures, mental health indicators, reproductive health factors, breastfeeding, physical fitness, and program feasibility metrics such as retention, satisfaction, and resource utilization.

Pregnancy provides a unique opportunity to identify women at increased risk of future chronic disease. It can be described as a “stress test,” unmasking predispositions to cardiometabolic dysfunction. Women who experience adverse pregnancy outcomes (APOs), such as hypertensive disorders of pregnancy (HDP), gestational diabetes (GD), preterm birth, placental abruption, or small‐for‐gestational‐age infants, face significantly elevated risks of later cardiovascular disease (CVD), type 2 diabetes, metabolic syndrome, and chronic kidney disease.^1^, ^2^ Despite this knowledge, postpartum care remains fragmented worldwide, even in high‐income health systems, and is often limited to a brief 6‐week clinical review. This represents a missed critical opportunity for preventive healthcare, with the FIGO (International Federation of Gynecology & Obstetrics) Committee on the Impact of Pregnancy on Long‐Term Health recently adapting a pregnancy passport. This innovative tool facilitates screening and planning for the optimal management of women who have experienced complications during pregnancy.^3^

Evidence shows that women with HDPs are twice as likely to develop chronic hypertension, CVD, and kidney disease, and those with GD have a seven‐fold increased risk of type 2 diabetes and a two‐fold increased risk of CVD within a decade of delivery.^1^, ^2^, ^4^ Without intervention, up to one in four women with GD progress to metabolic syndrome within 5 years after delivery.^5^ A major barrier is low uptake of follow‐up care, with fewer than half of high‐risk women attending postpartum checkups.^6^ Additional challenges include lack of resources, inconsistent communication, and limited integration with community services. Lifestyle programs—particularly those focusing on diet and exercise—can significantly reduce these risks. Pragmatic, locally specific programs are urgently needed.

We developed NuViva, a community‐integrated postpartum program combining cardiometabolic risk monitoring, nutrition and exercise interventions, pelvic floor rehabilitation, psychosocial support, and digital health tools. Designed by a multidisciplinary team, NuViva is currently being piloted.

Women eligible for NuViva are aged ≥18 years, have experienced APOs (e.g. GD, HDPs, small‐for‐gestational‐age infants, placental abruption, preterm birth), delivered at the local tertiary hospital, intend to enroll for 1 year, understand the local language, and provide written consent. Exclusions include preexisting hypertension or diabetes, medical advice against exercise, plans to relocate within 1 year, psychiatric or substance‐use hospitalization in the past year, and active eating disorders. Participants are enrolled postpartum after staff review medical records for eligibility assessment and confirmation.

NuViva provides 1 year of structured follow‐up, beginning with an assessment at 6 weeks postpartum and followed by four community‐based visits at 8 weeks, 3 months, 6 months, and 12 months (Figure 1). At each structured visit lasting up to 60 min, women undergo a comprehensive physical assessment measuring blood pressure, biometrics, and pelvic floor function. Where indicated, investigations may include hemoglobin, oral glucose tolerance test, renal function, urine protein–creatinine ratio, hemoglobin A_1c_, and/or lipid profile. Small group sessions of a maximum of four participants cover supervised exercises, nutrition counseling, and lactation support. Physical function is assessed using the 6‐min walk test, grip strength, standing vertical jump, and pelvic floor lift. Psychosocial and lifestyle assessments include the International Physical Activity Questionnaire (IPAQ), 24‐h dietary recall, the Edinburgh Postnatal Depression Scale (EPDS), General Anxiety Disorder‐7 (GAD‐7), the State–Trait Anxiety Inventory (STAI), and the Pittsburgh Sleep Quality Index (PSQI).

**FIGURE 1:**
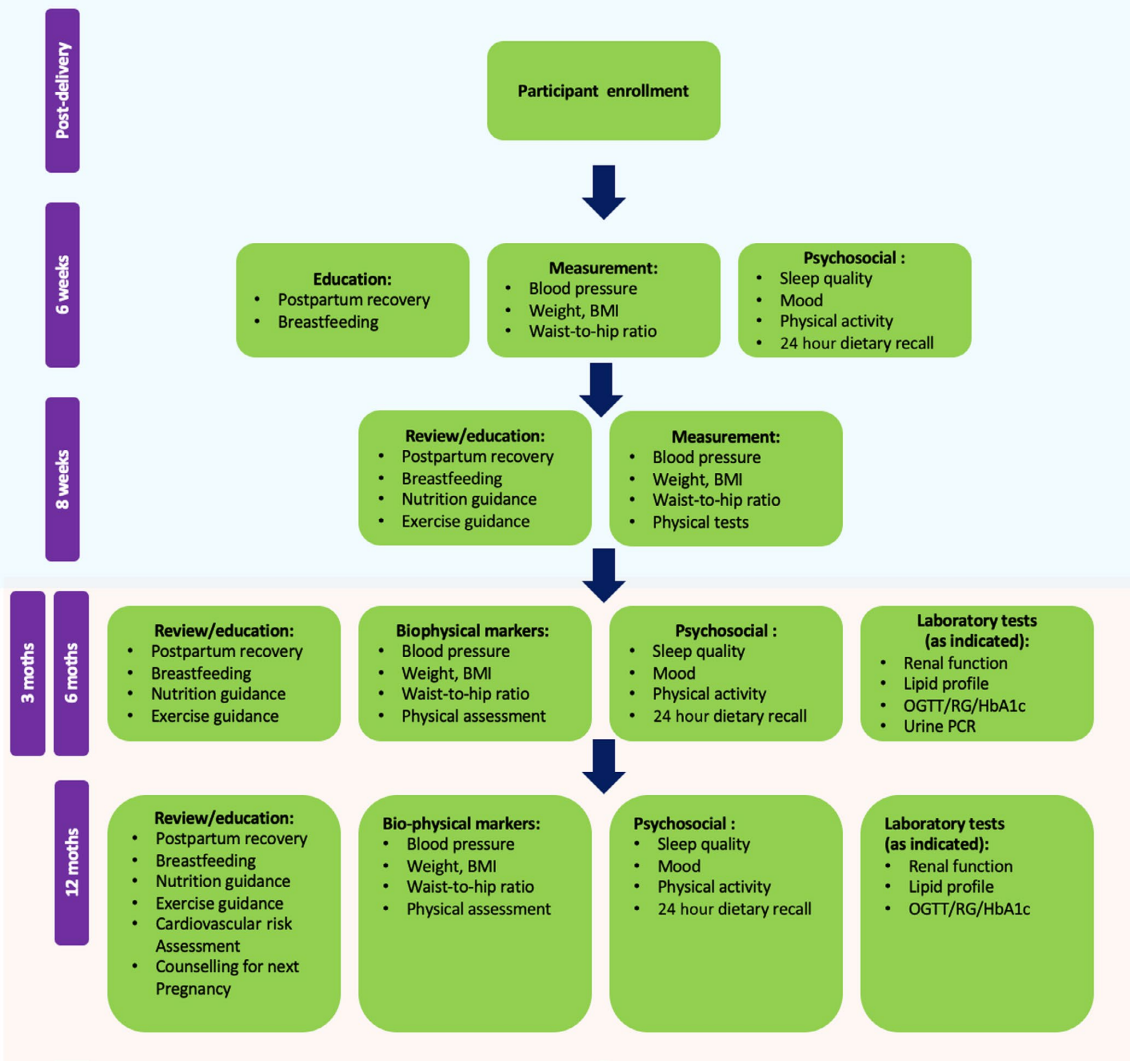
Flow diagram of the NuViva postpartum nutrition and exercise program 0 to 12 months. BMI, body mass index; HbA1c, hemoglobin A_1c_; OGTT, oral glucose tolerance test; PCR, protein–creatine ratio; RG, random glucose; Urine PCR, urine protein‐creatinine ratio.

The primary outcome is cardiovascular health score at 1 year postpartum, based on eight metrics: smoking, physical activity, diet, sleep quality, body mass index, blood pressure, lipids, and hemoglobin A_1c_. Secondary outcomes include hip and waist circumference, depression and anxiety, pregnancy intention, contraception, and breastfeeding. Physical fitness will be assessed using the 6‐min walk test, handgrip strength, and vertical jump tests. Program outcomes will evaluate retention and dropout reasons, app use, patient satisfaction, and provider time burden during follow‐up per participant in routine care.

We have also developed the NuViva smartphone app, following the recommendations of the FIGO Pregnancy Passport. It is designed to empower women in self‐monitoring and enhance adherence. Participants can input and monitor biometric data, complete psychosocial assessments, log lifestyle behaviors, and access educational resources and demonstration videos for safe postpartum exercises. Participants also receive personalized health reports and reminders for follow‐up visits.

NuViva is an implementation of the FIGO Pregnancy Passport, translating global recommendations into local solutions. NuViva is expected to provide benefits among several domains: cardiometabolic health through lifestyle counseling and education, mental health through screening, pelvic floor function through specialist physical therapy, and interpregnancy optimization, as healthier women are more likely to achieve improved outcomes in subsequent pregnancies, reducing recurrence of APOs. NuViva is a replicable model for other regions seeking to reduce the growing burden of maternal cardiometabolic disease.

The postpartum period should not represent the end of maternity care but rather the beginning of long‐term disease prevention. Currently being piloted in Hong Kong, NuViva may provide valuable insights into feasibility and benefits, paving the way for broader implementation and policy integration. By investing in women's health during pregnancy intervals, we reduce cardiometabolic morbidity, support mental and pelvic health, and create healthier conditions for future pregnancies.

## AUTHOR CONTRIBUTIONS

NMWL and LCP were responsible for the concept and design of the project. PHYL was responsible for the design of the physical exercise component and training staff to deliver the project. KYT was responsible for designing the nutrition component and training staff to deliver the project. NMWL drafted the manuscript. All other authors contributed to the final manuscript. LCP supervises the project.

## FUNDING INFORMATION

The authors have nothing to report.

## CONFLICT OF INTEREST STATEMENT

Nikki M. W. Lee, So‐Ling C. Lau, and Liona C. Poon have no conflicts of interest. Panda Hoi Yin Li is Founder and Director of Physiomotion Limited. Kit Ying Tsoi is currently an employee of Danone Nutricia Early Life Nutrition (Hong Kong) Limited.

## PATIENT CONSENT

Not applicable.

## Data Availability

Data sharing is not applicable to this article as no new data were created or analyzed in this study.
